# Durability of glucose-lowering effect of dulaglutide in patients with type 2 diabetes mellitus: A real-world data study

**DOI:** 10.3389/fendo.2022.1032793

**Published:** 2022-10-31

**Authors:** Hwi Seung Kim, Yun Kyung Cho, Myung Jin Kim, Chang Hee Jung, Joong-Yeol Park, Woo Je Lee

**Affiliations:** ^1^ Department of Internal Medicine, Chung-Ang University Gwangmyeong Hospital, Chung-Ang University College of Medicine, Gwangmyeong, South Korea; ^2^ Department of Internal Medicine, Asan Medical Center, University of Ulsan College of Medicine, Seoul, South Korea; ^3^ Asan Diabetes Center, Asan Medical Center, Seoul, South Korea

**Keywords:** type 2 diabetes mellitus, adherence, dulaglutide, real-world evidence, GLP-1 receptor agonist

## Abstract

**Introduction:**

Type 2 diabetes mellitus (T2DM) is a chronic, progressive disease requiring lifelong treatment, and durable medication is essential for maintaining stable glycemic control. This study aimed to evaluate the long-term efficacy of dulaglutide in participants who have continued the drug for more than one year.

**Methods:**

We conducted a retrospective study on 605 participants, who used dulaglutide for over one year between 2016 and 2020. Changes in glycosylated hemoglobin (HbA1c), fasting plasma glucose, and bodyweight from baseline to last prescription day were assessed. Adherence was evaluated by the proportion of days covered (PDC), and a PDC value ≥ 0.80 was considered adherent.

**Results:**

The mean age was 54.0 ± 11.1 years, and 46.1% were female. The mean baseline HbA1c, bodyweight, and duration of diabetes were 8.8% (72.7 mmol/mol), 75.6 kg, and 12.2 years, respectively. During the mean follow-up of 33.1 months, HbA1c and bodyweight decreased by 1.28% (14 mmol/mol, P < 0.001) and by 3.19 kg (P < 0.001), respectively. The participants were highly adherent with PDC ≥ 0.80 in 92.4% of the participants.

**Conclusion:**

In T2DM patients, long-term dulaglutide treatment was effective in maintaining HbA1c and weight reduction. Dulaglutide could be a favorable option of long-term treatment in real-world clinical practice.

## Introduction

Type 2 Diabetes Mellitus (T2DM) is a chronic progressive disease affecting nearly 10% of the adult population worldwide (1). The extent of glycemic control is strongly associated with diabetic complications and mortality (2). Therefore, maintenance of the glycemic goal is critical for patients with T2DM. Antidiabetic drugs show variable durability. Thiazolidinedione has been associated with the most durable glycemic response, followed by sulfonylurea and dipeptidyl-peptidase-4 inhibitor (3). Also, sodium-glucose co-transporter 2 inhibitor show greater durability than dipeptidyl-peptidase-4 inhibitor (4).

The control rate of diabetes is low with only about half of the US diabetic population reaching the target glycosylated hemoglobin (HbA1c) of < 7.0% (5, 6). For patients who do not reach the goal, intensification of medical treatment is often necessary. For those who achieve the target, sustenance of the glycemic control is essential. Although it should be supported by diet and exercise, medical therapy is of great importance. In the last decade, the novel drug class glucagon-like peptide-1 receptor agonist (GLP-1RA) has been introduced. Due to its additional effects on weight loss and low risk of hypoglycemia, GLP-1RA use has been increasing continuously (7). Among the various currently available GLP-1RAs, dulaglutide or semaglutide is preferred because of the convenience of once-weekly administration (8–10).

Dulaglutide has been associated with significant reductions in HbA1c and bodyweight as well as cardiovascular benefits in the Assessment of Weekly Administration of Dulaglutide in Diabetes (AWARD) and Researching Cardiovascular Events With a Weekly Incretin in Diabetes (REWIND) trials (11, 12). Following the actual clinical use of dulaglutide, retrospective real-world studies were also published globally, confirming the efficacy of dulaglutide. However, in most studies, the duration of drug usage was quite short (13–15). Since diabetes is a chronic and progressive disease, it is important to maintain glycemic control for a long period of time (16). Although a few studies did confirm the long-term efficacy of dulaglutide, there has been no report, to our knowledge, on the long-term efficacy of dulaglutide in Asian patients. Therefore, we conducted this real-world study to investigate the durability of dulaglutide treatment in Korean patients with T2DM.

## Material and methods

### Participants

**Figure 1:**
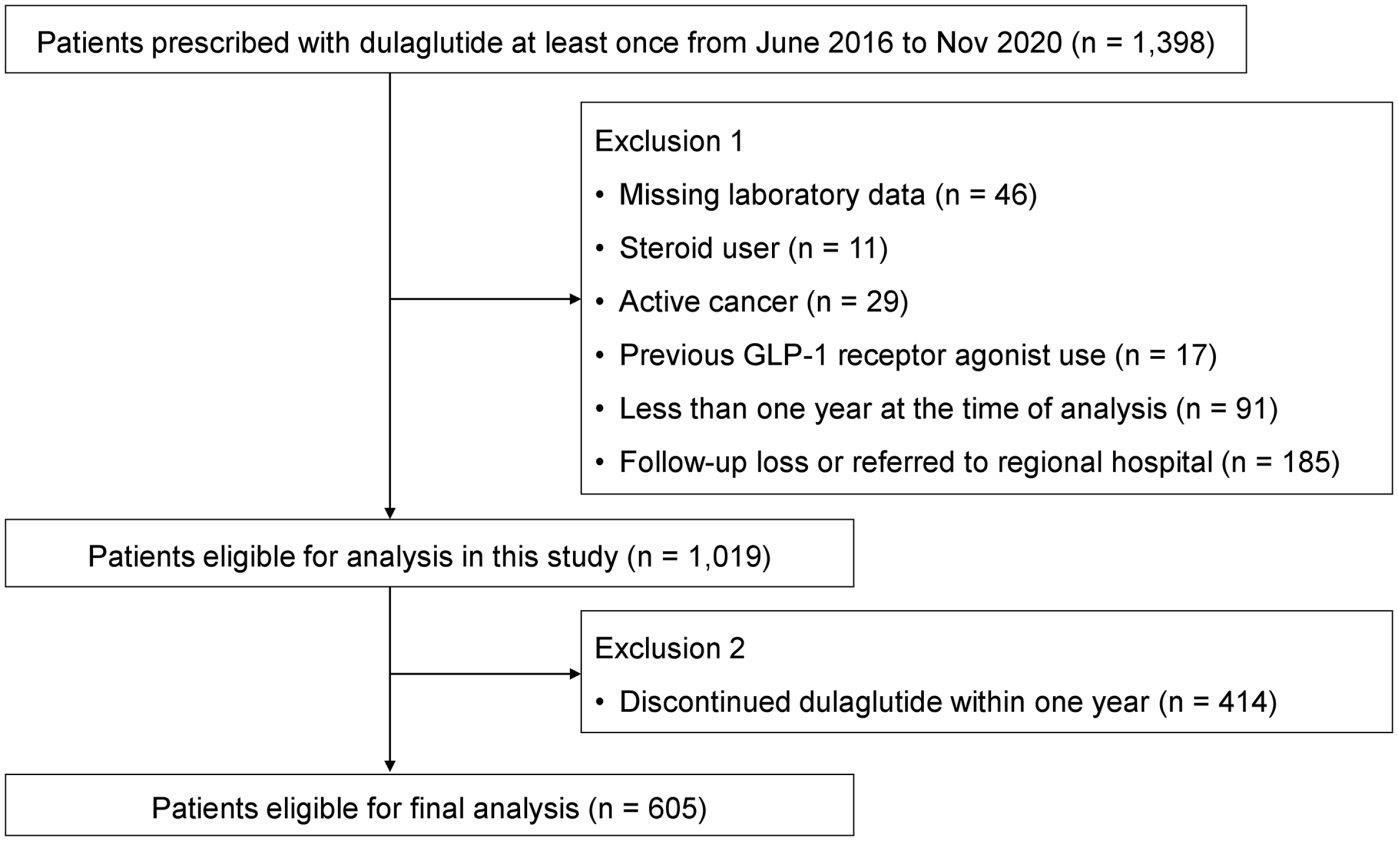
Flow diagram showing the selection process of the study population for durability of dulaglutide.

We initially identified 1398 T2DM patients who were prescribed with dulaglutide at least once from June 2016 to November 2020 (**Figure 1**). After thorough review of medical records, patients with missing laboratory data (n = 46), steroid use (n = 11), active cancer (n = 29), and previous GLP-1RA use (n = 17) were excluded. We further excluded patients who had been using dulaglutide for less than a year by the time of analysis (n = 91) and those who were not present for follow-up or were referred to regional hospitals (n = 185). Of the 1019 patients eligible for analysis, 605 patients continued dulaglutide treatment for over one year and were included in the final analysis. Minimum 12-month use was suggested for an anti-diabetic drug to be considered durable (17). This study followed the principles of the Declaration of Helsinki and Korean Good Clinical Practice and was approved by the Institutional Review Board of Asan Medical Center (IRB No. 2020-1914).

### Clinical and laboratory measurements

Clinical data including age, sex, bodyweight, height, body mass index (BMI), blood pressure (BP), duration of diabetes, co-existing hypertension, dyslipidemia, diabetic complications, and use of other medications were collected. For participants who had not continued dulaglutide treatment for a year, the reasons for discontinuation were documented. Laboratory measurements consisted of glycosylated hemoglobin (HbA1c), fasting plasma glucose (FPG), total cholesterol (TC), triglycerides (TG), high-density lipoprotein cholesterol (HDL-C), low-density lipoprotein cholesterol (LDL-C), aspartate aminotransferase (AST), alanine aminotransferase (ALT), creatinine, estimated glomerular filtration rate (eGFR), and fasting c-peptide. Data on concomitant anti-diabetic drugs, HbA1c, FPG, and weight on the last prescription day were assembled.

### Outcomes

The primary outcome measure was the evaluation of glycemic control in participants who initiated dulaglutide treatment and continued for at least one year. For this purpose, change of HbA1c from baseline to last prescription day was measured. Additionally, HbA1c, fasting plasma glucose (FPG), and bodyweight were also assessed every six months. The secondary outcome measure was adherence, which was measured by the proportion of days covered (PDC), evaluated by chart review. PDC was calculated as the total number of days covered by prescribed dulaglutide divided by the total number of days in the post-initiation follow-up period (13). Participants with a PDC of 0.80 or higher were considered adherent (13). Subgroup analyses of HbA1c reduction according to age, baseline HbA1c, BMI, duration of diabetes, starting dulaglutide dose, and final dulaglutide dose were performed. Subgroup analyses of HbA1c and weight reduction according to baseline antidiabetic drug use and change in other antidiabetic drugs were also conducted.

### Statistical analysis

Continuous and categorical variables were each shown as mean ± standard deviation and number (percentages), respectively. Changes in HbA1c, FPG, and bodyweight over time were analyzed using a repeated measures model. Paired t-tests were performed for subgroup analyses. To evaluate the parameters affecting the glycemic response, univariate and multivariate linear regression analyses were performed. All statistical analyses were performed using SPSS version 23.0 for Windows (IBM Co., Armonk, NY, USA).

## Results

### Selected population and baseline characteristics

A total of 1019 participants were eligible for analysis after applying the exclusion criteria (**Figure 1**). Of these, 414 participants were further excluded because of discontinuation of dulaglutide within a year after initiation. As a result, 605 participants who continued dulaglutide for more than one year (mean follow-up 33.1 months) were included in the final analyses. The reasons for discontinued dulaglutide use are presented in **Table 1**. The most common reason for stopping dulaglutide was adverse events (160 participants; 38.6%). Among the 160 participants who discontinued the drug due to adverse events, 139 experienced gastrointestinal trouble, of which nausea was by far the most experienced by 108 participants. Eighteen and three participants stopped the drug due to injection site problem and hypoglycemia, respectively. Other reasons for discontinuing dulaglutide were the use of injection as the mode of administration (67 participants; 16.2%), poor glycemic control (123 participants; 29.7%), and good glycemic control (8 participants; 1.9%). Decision to discontinue dulaglutide for poor or good glycemic control was based on the clinician’s judgment. All patients who stopped dulaglutide due to poor glycemic control were switched to insulin. Patients who reached the individualized HbA1c target and de-escalated treatment by discontinuing dulaglutide were considered good glycemic control. Three deaths were identified, and the causes of death were stroke and pneumonia for two and unknown for one.

**Table 1:** Reasons for discontinuing dulaglutide within one year.

**Total number of patients available for analysis**	1019
**Continued dulaglutide for more than one year**	605 (59.4)
**Discontinued dulaglutide within one year**	414 (40.6)
**Adverse events**	160 (38.6)
GI trouble	139 (33.6)
Nausea	108 (26.1)
Vomiting	4 (1.0)
Diarrhea	11 (2.7)
Anorexia	13 (3.1)
Abdominal pain	3 (0.7)
Hypoglycemia	3 (0.7)
Injection site problem	18 (4.3)
**Good glycemic control**	8 (1.9)
**Poor glycemic control**	123 (29.7)
**Too much weight loss**	1 (0.2)
**Rejected injection**	67 (16.2)
**Switched to anti-obesity drug**	14 (3.4)
**Gastrectomy**	3 (0.7)
**Pregnancy**	2 (0.5)
**In hospital care for surgery and infection**	15 (3.6)
**Death**	3 (0.7)
**Unknown reason**	18 (4.3)

Values are presented as number (%).

Baseline characteristics of the 605 participants who continued dulaglutide for over one year are shown in **Table 2**. The mean age was 53.96 ± 11.12 years, and 46.2% of the participants were female. The mean bodyweight was 75.63 kg, and the mean BMI was 27.99 kg/m^2^. The mean duration of diabetes was 12.23 years. HbA1c and FPG levels were 8.8% and 184.94 mg/dL, respectively. The mean fasting c-peptide concentration was 2.60 ng/dL. Hypertension and dyslipidemia were present in 66.0% and 89.9% of the study population, respectively.

**Table 2:** Baseline characteristics of the study population.

Characteristic, unit	Mean ± SD, Number (%)
Age, yr	53.96 ± 11.12
Female	279 (46.1)
Bodyweight, kg	75.63 ± 14.82
Body mass index, kg/m^2^	27.99 ± 5.34
Duration of diabetes, yr	12.23 ± 14.03
HbA1c, %	8.8 ± 1.7
Fasting plasma glucose, mg/dL	184.94 ± 61.94
Systolic blood pressure, mmHg	130.66 ± 16.59
Diastolic blood pressure, mmHg	75.68 ± 11.33
Creatinine, mg/dL	0.87 ± 0.33
Estimated GFR, mL/min/1.73 m^2^	90.38 ± 21.88
Aspartate transaminase, IU/L	27.86 ± 16.24
Alanine transferase, IU/L	28.98 ± 20.23
Total cholesterol, mg/dL	155.02 ± 71.95
Triglycerides, mg/dL	184.23 ± 177.47
High-density lipoprotein cholesterol, mg/dL	45.70 ± 11.20
Low-density lipoprotein cholesterol, mg/dL	97.90 ± 56.34
C-peptide, ng/dL	2.60 ± 1.46
Hypertension	399 (66.0)
Dyslipidemia	544 (89.9)
Diabetes complications	
Retinopathy	233 (38.5)
Nephropathy	227 (37.5)
Neuropathy	102 (16.9)
Cardiovascular disease	74 (12.2)
Cerebrovascular disease	26 (4.3)
Peripheral vascular disease	5 (0.8)

Values are presented as mean ± standard deviation or number (%). GFR, glomerular filtration rate.

### Treatment patterns

Majority of the participants (555 of 605; 91.7%) started the treatment with 0.75 mg of dulaglutide, and 83.4% of these participants switched to 1.5 mg of dulaglutide during follow-up. Of the 50 (8.3%) participants who initiated dulaglutide use at 1.5 mg, only one patient decreased the dose to 0.75 mg during follow-up.

All the participants were on at least one oral antihyperglycemic drug at baseline (**Table 3** **).** Metformin was the most common oral antihyperglycemic drug used by the participants at baseline (592 participants; 97.9%), followed by sulfonylurea (487 participants; 80.5%). Insulin was used by 138 participants (22.8%). At the last follow-up visit, the number of participants using metformin and sulfonylurea decreased to 579 (95.7%) and 426 (70.4%), respectively. Conversely, the use of thiazolidinedione, a sodium-glucose co-transporter 2 (SGLT2) inhibitor, and insulin increased. Number of participants using SGLT2 inhibitor showed the greatest increase from 63 (10.4%) at baseline to 141 (23.3%) at last follow-up.

**Table 3:** Use of concomitant anti-diabetic medication with dulaglutide at baseline and last follow-up.

Medication	Baseline	Last Follow-up
Metformin	592 (97.9)	579 (95.7)
Sulfonylurea	487 (80.5)	426 (70.4)
Thiazolidinedione	7 (1.2)	20 (3.3)
SGLT2 inhibitor	63 (10.4)	141 (23.3)
Any Insulin	138 (22.8)	145 (24.0)

Values are presented as number (%). SGLT2, sodium-glucose co-transporter 2.

### Efficacy of long-term dulaglutide

**Figure 2:**
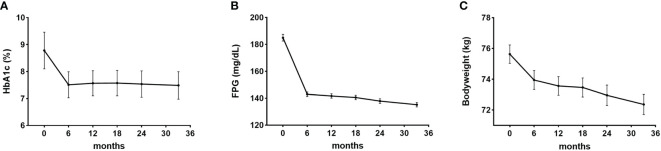
Efficacy of dulaglutide. Changes in **(A)** glycosylated hemoglobin (HbA1c), **(B)** fasting plasma glucose (FPG), and **(C)** bodyweight after dulaglutide use. Data are presented as mean ± standard error. All values for 6–36 months in each of the panels **(A–C)** were significantly different (P < 0.05) compared with the corresponding baseline values.

HbA1c levels reduced significantly after using dulaglutide for six months and were maintained through follow-up (P < 0.001) (**Figure 2A**). Mean reduction in HbA1c was 1.2 ± 1.6% from baseline to last follow-up. FPG levels also significantly decreased after six months of dulaglutide use and were maintained until last follow-up (P < 0.001) (**Figure 2B**). Mean FPG decrease was 46.1 ± 59.5 mg/dL. Bodyweight was significantly reduced by 3.3 ± 5.4 kg from baseline to last follow-up (P < 0.001) (**Figure 2C**). The ratio of participants with PDC ≥ 0.80 was 92.4%, and the mean PDC was 0.98.

### Clinical parameters affecting the glucose-lowering effect of long-term dulaglutide

**Figure 3:**
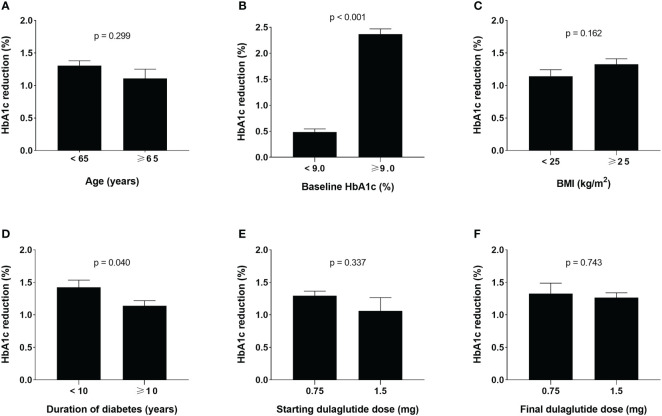
Glucose-lowering effects of dulaglutide among subgroups. Subgroup analyses for changes in HbA1c levels according to **(A)** age, **(B)** baseline HbA1c, **(C)** BMI, **(D)** duration of diabetes, **(E)** initial dulaglutide dose, and **(F)** final dulaglutide dose. Data are presented as mean ± standard error. HbA1c, glycosylated hemoglobin; BMI, body mass index.

According to univariate linear regression analysis, factors predicting the glucose-lowering efficacy of long-term dulaglutide treatment were baseline HbA1c and baseline FPG levels (**Table 4**). Multiple linear regression analysis showed that duration of diabetes and baseline HbA1c levels significantly affected HbA1c reduction (**Table 5**). Subgroups divided by baseline HbA1c levels (< 9.0% versus ≥ 9.0%) and diabetes duration (< 10 years versus ≥ 10 years) showed significant differences in the levels of HbA1c reduction; participants with higher baseline HbA1c levels and shorter diabetes durations responded better to dulaglutide. However, no significant difference in HbA1c reduction was observed between the subgroups defined according to age, BMI, or initial/final dulaglutide dose (**Figure 3**). In all subgroups regarding other antidiabetic drug use at baseline, significant decreases in HbA1c and bodyweight were observed (**Supplemental Table 1**). When the subjects were divided according to change in other antidiabetic drugs, HbA1c and bodyweight also showed significant reduction (**Supplemental Table 2**).

**Table 4:** Univariate linear regression analysis of HbA1c reduction after dulaglutide use.

Variable	Standardized b	P value
Age	–0.021	0.614
Sex	–0.020	0.622
Baseline bodyweight	0.049	0.233
Body mass index	0.055	0.175
Duration of diabetes	–0.050	0.216
Baseline HbA1c	0.748	< 0.001
Baseline FPG	0.400	< 0.001
Baseline c-peptide	0.016	0.712
Hypertension	–0.073	0.071
Dyslipidemia	–0.022	0.595
Initial dulaglutide dose	–0.037	0.366
PDC	–0.037	0.370

HbA1c, glycosylated hemoglobin; FPG, fasting plasma glucose; PDC, proportions of days covered.

**Table 5:** Multiple linear regression analysis of HbA1c reduction after dulaglutide use.

Variable	Standardized b	P value
Baseline bodyweight	–0.012	0.742
Body mass index	0.001	0.985
Duration of diabetes	–0.069	0.014
Baseline HbA1c	0.763	< 0.001
Baseline FPG	–0.025	0.444
Hypertension	–0.038	0.168

HbA1c, glycosylated hemoglobin; FPG, fasting plasma glucose.

## Discussion

Our results showed that long-term dulaglutide treatment was effective and safe in patients with T2DM. In real-world clinical practice, dulaglutide significantly improved glycemic control in the first six months, and this effect was maintained throughout the period of dulaglutide treatment. In parallel, FPG and bodyweight also showed similar patterns of sustainable improvement. Higher baseline HbA1c levels and shorter duration of diabetes were associated with greater reductions in HbA1c levels.

While most studies on dulaglutide evaluated its effectiveness over a period of 6–12 months, two previous retrospective studies evaluated its effects after 2 years of usage. Moreno Obregón et al. analyzed 163 Spanish patients and found 1.4% (15 mmol/mol) reduction in HbA1c (P < 0.001) and 30 mg/dL reduction in FPG levels (P < 0.001) after 6 months of drug use, both of which were maintained until the follow-up at 2 years (18). Bodyweight, evaluated every 6 months, showed a continues decrease, resulting in a reduction of 7.27 kg after 2 years (P < 0.001). Compared with our results, the study on Spanish patients reported a greater reduction in HbA1c, a smaller decrease in FPG, and almost 4 kg greater reduction in bodyweight. This might be explained by the difference in the baseline bodyweight (75.63 kg in our study population versus 99.57 kg in the Spanish study population). Another study recently conducted in the United States (US) evaluated the efficacy of dulaglutide over a period of 2 years in 872 patients (19). The baseline HbA1c level was 8.7%, similar to that 8.8% in our study. The extent of HbA1c reduction was 1.3% and 1.2% in the US study and the current study, respectively. Unfortunately, the US study did not report data on weight reduction or percentage of Asian ethnic group included in the study. So, we cannot make any comparisons in these aspects. However, subgroup analyses of both studies showed that HbA1c levels reduced regardless of sex, age, and index dulaglutide dose, which is also accordant with the results of *post-hoc* analysis of the AWARD trials (20). The patterns of other antihyperglycemic medication use at baseline and at 24-month follow-up in the US study were similar to those in our study with increased use of SGLT2 inhibitor, thiazolidinedione, and insulin and decreased use of sulfonylurea. Previous reports of GLP-1RA and SGLT2 inhibitor combination therapy suggest significant weight reduction benefits and possible additional cardiovascular benefits, which could explain their increased use (21–23).

Results of our analysis comparing BMI subgroups did not differ from those of the *post-hoc* analysis of the AWARD trials, showing no significant differences in HbA1c reduction levels among the subgroups (24). However, the mean baseline BMI levels in the AWARD trials, ranging between 31.2 and 33.3 kg/m^2^, were greater compared with the corresponding levels of 27.99 kg/m^2^ in our study. In addition to the disparity in the mean BMI values, the BMI categories were also defined by different intervals (<30 kg/m^2^; ≥30 and <35 kg/m^2^; ≥35 kg/m^2^ versus <25 kg/m^2^; ≥25 kg/m^2^). The use of different criteria for BMI categories seems reasonable, as the diagnostic criteria for obesity are different in East Asian (25). Another real-world study by Morieri et al. showed that improvements in HbA1c and weight indices were significant and consistent regardless of age, obesity, and chronic kidney disease, while these effects were greater in patients with shorter durations of diabetes and in those who had not used GLP-1RAs previously (26). Since the patients in our study were all GLP-1RA-naïve, we could not analyze the data to examine the effects of previous GLP-1RA exposure. Nevertheless, our study adds further evidence that shorter duration of diabetes is associated with a more pronounced HbA1c reduction.

The AWARD-5 trial evaluated the effects of long-term dulaglutide use (over 24 months) (27). HbA1c and bodyweight indices were significantly reduced by 1.0% and 2.9 kg, respectively, after 2 years (P < 0.001 for both) (27). The lower baseline HbA1c levels (8.1% vs 8.8% in our study) could have influenced the extent of reductions in HbA1c and bodyweight, which were smaller than those in our study (1.2% and 3.3 kg). Also, all patients in the AWARD-5 trial were on metformin as the only background hypoglycemic medication. Additionally, since AWARD-5 trial was a controlled randomized clinical trial, the patients who discontinued dulaglutide during the 2-year follow-up period were also included in the final analysis of the results.

Adherence is frequently suboptimal in T2DM patients, which can worsen glycemic control, increase hospitalization, and lead to diabetic complications (28). Mody et al. previously reported an adherence of 61% over a dulaglutide treatment period of 6 months with a mean PDC of 0.76 (13), which are substantially less than the 92% adherence and mean PDC of 0.98 in our study. The high adherence in our study might have ensued from selection bias because our analysis only included patients who continued dulaglutide treatment for more than one year. Also, since a total of 320 patients showed a PDC greater than 1.0, PDC might not completely reflect the actual administration of the drug. Additionally, Durden et al. showed that early response to GLP-1RA (defined as at least 1% reduction in HbA1c and at least 3% reduction in bodyweight within 3–6 months) is associated with higher adherence (29). The present study population showed mean reductions of 1.2% in HbA1c and 4.4% in bodyweight from baseline during the first 6 months of dulaglutide initiation, which might have contributed to the high adherence rate.

This study has some limitations. First, it is a retrospective study based on data acquired from chart review, so the adverse events and hypoglycemic episodes reported by patients could be under-estimated or over-estimated. Additionally, the retrospective nature of the study could have caused selection bias, and the patients excluded could have influenced the results. Second, the results might not be generalized, because the analyses were conducted on data obtained from a single institution. Third, the current study was not controlled, so other medications taken in parallel could have affected the results. Finally, while GLP-1RA is in the limelight for its pleiotropic benefits in addition to its glucose-lowering and weight-reducing effects, we have not assessed its cardiovascular or renal outcomes, which we plan to investigate in the near future.

While the retrospective, observational aspect of the current study might be a limitation, our results may provide valuable insights on the real-world clinical practice of T2DM management. As clinical trials follow a tightly defined criteria for patient enrollment, study populations in clinical trials may not be truly representative of general patient populations. In contrast, real-world evidence provides objective data about treatment trends and patient outcomes in general clinical practice. To the best of our knowledge, this is the first study to evaluate long-term efficacy of dulaglutide in Asian population in real world. In addition, relatively large number of participants were analyzed, and data on adherence and sustainability of dulaglutide were presented.

In summary, our study revealed that dulaglutide shows sustainable efficacy and safety in real-world clinical practice. HbA1c, FPG, and bodyweight indices were significantly improved after 6 months of dulaglutide use, and the improvements were maintained throughout the period of dulaglutide treatment. As the first real-world study of long-term dulaglutide treatment in Asia, this study contributes valuable data to the literature on glycemic effectiveness and sustainability of dulaglutide.

## Data availability statement

The raw data supporting the conclusions of this article will be made available by the authors, without undue reservation.

## Ethics statement

The studies involving human participants were reviewed and approved by Institutional Review Board of Asan Medical Center (IRB No. 2020-1914). Written informed consent for participation was not required for this study in accordance with the national legislation and the institutional requirements.

## Author contributions

WL designed the research; HK and MK conducted research; YC, CJ, WL, and J-YP provided essential materials; HK, MK, YC analyzed data and wrote paper; HK, YC, CJ, and WL had primary responsibility of the final content; All authors contributed to the article and approved the submitted version.

## Conflict of interest

The authors declare that the research was conducted in the absence of any commercial or financial relationships that could be construed as a potential conflict of interest.

## Publisher’s note

All claims expressed in this article are solely those of the authors and do not necessarily represent those of their affiliated organizations, or those of the publisher, the editors and the reviewers. Any product that may be evaluated in this article, or claim that may be made by its manufacturer, is not guaranteed or endorsed by the publisher.
