# Supplementary material for: Durability of glucose-lowering effect of dulaglutide in patients with type 2 diabetes mellitus: A real-world data study
Source: Front Endocrinol (Lausanne). 2022 Oct 31;13:1032793. doi: 10.3389/fendo.2022.1032793 (PMC9659594; doi:10.3389/fendo.2022.1032793)
Supplement: Supplementary file 1 [file DataSheet_1.docx]

**Supplementary Material**

**Supplemental Table 1. Glycemic control after dulaglutide treatment among subgroups according to concurrent antidiabetic drugs at baseline**

| **Subgroup** | **Number of patients** | **Baseline HbA1c, mean (SD), %** | **HbA1c change, mean (SD), %** | **P value** | **Baseline bodyweight, mean (SD), kg** | **Bodyweight change, mean (SD), kg** | **P value** |
| --- | --- | --- | --- | --- | --- | --- | --- |
| **Metformin** |  |  |  |  |  |  |  |
| Yes | 592 | 8.8 (1.7) | –1.3 (1.6)* | 0.12 | 75.7 (14.9) | –3.2 (5.2)* | 0.501 |
| No | 13 | 8.7 (2.2) | –2.0 (2.0)* |  | 70.9 (12.3) | –4.1 (4.6)* |  |
| **Sulfonylurea** |  |  |  |  |  |  |  |
| Yes | 487 | 8.8 (1.6) | –1.2 (1.6)* | 0.30 | 75.0 (14.8) | –3.1 (5.1)* | 0.46 |
| No | 118 | 8.8 (2.0) | –1.6 (1.9)* |  | 78.4 (14.8) | –3.5 (5.3)* |  |
| **SGLT2 inhibitor** |  |  |  |  |  |  |  |
| Yes | 63 | 9.1 (1.8) | –1.3 (1.6)* | 0.95 | 82.4 (15.2) | –3.0 (3.8)* | 0.72 |
| No | 542 | 8.7 (1.6) | –1.3 (1.7)* |  | 74.8 (14.6) | –3.2 (5.3)* |  |

HbA1c, glycosylated hemoglobin; SD, standard deviation; SGLT2, sodium-glucose cotransporter 2.

* P < 0.01 from paired t test.

**Supplemental Table 2. Glycemic control after dulaglutide treatment among subgroups according to change in other antidiabetic drugs**

|  | **Number of patients** | **Baseline HbA1c, mean (SD), %** | **HbA1c change, mean (SD), %** | **Baseline bodyweight, mean (SD), kg** | **Bodyweight change, mean (SD), kg** |
| --- | --- | --- | --- | --- | --- |
| Change | 267 | 8.9 (1.9) | –1.5 (1.6)* | 78.9 (15.0) | –3.9 (6.4)* |
| No Change | 338 | 8.7 (1.5) | –1.1 (2.0)* | 73.2 (14.2) | –2.6 (3.8)* |

HbA1c, glycosylated hemoglobin; SD, standard deviation.

* P < 0.01 from paired t test..
